# Mediation Effect of Kinesiophobia on the Relationship between Cervical Joint Position Sense and Limits of Stability in Individuals with Fibromyalgia Syndrome: A Cross-Sectional Study Using Mediation Analysis

**DOI:** 10.3390/jcm12082791

**Published:** 2023-04-09

**Authors:** Mastour Saeed Alshahrani, Ravi Shankar Reddy

**Affiliations:** Department of Medical Rehabilitation Sciences, College of Applied Medical Sciences, King Khalid University, Abha 61421, Saudi Arabia; msdalshahrani@kku.edu.sa

**Keywords:** joint position sense, fibromyalgia, limits of stability, postural control, kinaesthesis, chronic pain

## Abstract

(1) Background: Individuals with fibromyalgia syndrome (FMS) may experience proprioceptive and balance impairments. Kinesiophobia is a factor that can mediate the relationship between cervical joint position sense (JPS) and limits of stability. The objectives of this study were to (1) compare the cervical JPS and limits of stability between FMS and asymptomatic individuals, (2) assess the relationship between cervical JPS and limits of stability, and (3) assess the mediation effect of kinesiophobia on the relationship between cervical JPS and limits of stability in FMS individuals. (2) Methods: In this comparative cross-sectional study, 100 individuals with FMS and 100 asymptomatic individuals were recruited. Cervical JPS was assessed using a cervical range of motion device, limits of stability (reaction time, maximum excursion, and direction control) were assessed using dynamic posturography, and FMS individuals’ kinesiophobia was assessed using the Tampa scale of kinesiophobia (TSK). Comparison, correlation, and mediation analyses were performed. (3) Results: The magnitude of the mean cervical joint position error (JPE) was significantly larger in FMS individuals (*p* < 0.001) compared to the asymptomatic individuals. The limits of the stability test showed that FMS individuals had a longer reaction time (F = 128.74) and reduced maximum excursion (F = 976.75) and direction control (F = 396.49) compared to the asymptomatic individuals. Cervical JPE showed statistically significant moderate-to-strong correlations with reaction time (r = 0.56 to 0.64, *p* < 0.001), maximum excursion (r = −0.71 to −0.74, *p* < 0.001), and direction control (r = −0.66 to −0.68, *p* < 0.001) parameters of the limits of the stability test. (4) Conclusions: Cervical JPS and limits of stability were impaired in FMS individuals, and the cervical JPS showed a strong relationship with limits of stability variables. Moreover, kinesiophobia mediated the relationship between JPS and limits of stability. These factors may be taken into consideration when evaluating and developing treatment strategies for FMS patients.

## 1. Introduction

Fibromyalgia syndrome (FMS) is a rheumatological disorder of widespread musculoskeletal pain chronic in nature, leading to significant disability [1]. These patients present with multiple sites of pain or tender points, fatigue, anxiety, poor sleep, depression, and emotional or mood fluctuations, resulting in work absence and rising healthcare costs [2,3]. The prevalence of FMS among females (9.3%) was found to be significantly higher (*p* < 0.001) than that among males (3.1%). In addition, participants aged 40 years old or more showed a significantly higher prevalence of FMS (*p* = 0.003) compared to those aged less than 40 years old (11.7% versus 6.0%, respectively) [4]. Balance control consists of several neural subsystems that may be affected by FMS. Balance issues were listed as one of the top 10 most incapacitating symptoms in FMS individuals [5].

The process of keeping one’s postural equilibrium and postural stability involves the integration of multiple systems [6]. Limits of stability is important in maintaining static and dynamic balance, and impairment will lead to falls in FMS individuals [7,8]. The literature has shown a moderate-to-strong association between impaired postural control and increased falls [9,10]. Johansson et al. demonstrated that the amplitude of postural sway in mm^2^ could significantly predict falls in the elderly, representing a functional balance [11]. Therefore, a thorough understanding of the systems responsible for maintaining postural balance is necessary for preventing falls and evaluating fall risks. Different authors have shown a relationship between decreased proprioceptive ability in peripheral joints and altered functional balance [12,13].

Cervical joint position sense (JPS) is vital in marinating head orientation and strength in relation to the trunk and providing efficient neuromuscular movement control of the cervical spine [14]. Cervical JPS is mediated by the integrated function of proprioceptors present in the muscles, capsules, joints, and vestibular and visual stimuli. Joint stability is significantly influenced by sensory information from mechanoreceptors in structures in and around the joints [15,16]. Cervical joint position sense (JPS) significantly contributes to the functional balance in FMS [17].

“Kinesiophobia” refers to the fear of movement brought on by activity or exercise and the catastrophic belief that such activity would result in damage or re-injury [18]. Kinesiophobia can cause mobility restriction in individuals with chronic pain and can change motor activation patterns [19]. These altered activation patterns can result in somato-motor-system-related muscular weakening and atrophy [20]. These modifications can significantly alter the afferent proprioceptive input, hence affecting the joint position sensation and stability limits [21]. Chronic persistent pain might exacerbate FMS patients’ fear of movement, hence diminishing their proprioceptive and balance abilities [21]. These findings demonstrate that kinesiophobia may serve as a mediator between cervical JPS and limitations of stability in patients with FMS. To date, there has been no evaluation of this mediation effect. We analyzed this relationship using a multiple regression model with mediation analysis. Understanding how kinesiophobia may impact the relationship between cervical JPS and limitations of stability is crucial for assessing and formulating treatment strategies for elderly adults with FMS. The study’s objectives are to (1) compare the cervical JPS and limits of stability between FMS individuals and asymptomatic, (2) assess the association between JPS and limits of stability, and (3) assess the mediation effect of kinesiophobia on the relationship between cervical JPS and limits of stability in FMS individuals. 

## 2. Materials and Methods

### 2.1. Study Design, Subjects, and Ethics

This study included 100 FMS individuals (mean age = 59.53 ± 7.72 years) and 100 asymptomatic individuals (mean age = 58.30 ± 4.23 years). The rheumatologist diagnosed the FMS patients according to the American College of Rheumatology criteria. The FMS individuals were included if they are over 50 years and met the diagnostic criteria. The FMS individuals were excluded if they had a history of dizziness, cervical disc disease, signs and symptoms of radiculopathy, or cervical disc disease. Subjects with a positive Spurling test indicated a diagnosis of cervical radiculopathy [22]. The signs and symptoms of disc disease included a sharp pain that radiates from the neck to the shoulder, arms, hands, and fingers; muscle weakness; numbness; spasms; headaches; and neck stiffness. The asymptomatic subjects were recruited from the university campus and neighborhood in the local community. The asymptomatic participants were included if they were physically fit, had no musculoskeletal complaints, and had no injuries. Each participant provided informed consent before the commencement of this study. This research study followed guidelines laid down by the Declaration of Helsinki. The KKU ethical committee approved this study (ECM# 2022-19-08). 

### 2.2. Kinesiophobia

Fear of movement experienced by FMS individuals was assessed using the Tampa scale of kinesiophobia (TSK), a reliable tool to estimate the magnitude of kinesiophobia experienced by an individual [18]. There are 17 questions on the scale. When a question is answered, a rating of 1 (total disagreement) or 4 (strong agreement) is assigned (complete agreement). The total TSK score ranges from 17 to 68. If the result is higher than 37, kinesiophobia is probably present. The TSK has been verified and translated into Arabic, and researchers have discovered that the scale has adequate internal consistency (r= 0.87) and excellent reliability (r = 0.86) [23].

### 2.3. Cervical Joint Position Sense Evaluation

**Figure 1:**
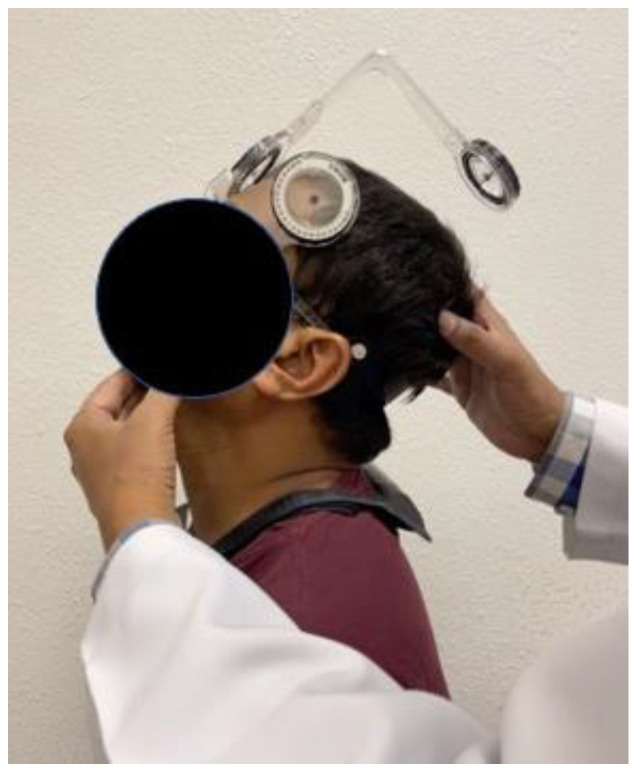
Cervical joint position sense evaluation using a CROM device.

The cervical JPS assessment was carried out using the cervical range of motion (CROM) tool (Performance Attainment Associates, Roseville, MN, USA) (Figure 1). 

CROM is a valid and reliable instrument for measuring cervical range of motion and JPS in individuals with and without neck symptoms [24,25,26]. The unit is helmet-shaped, including three inclinometers to measure the range of motion in three planes (frontal, sagittal, and transverse). The unit comprises a magnetic yoke specifically utilized to limit the influence of thoracic rotation during right and left rotation measurements. The target head position sense was evaluated as a measure of the cervical JPS [27]. The target was 25 degrees of flexion, extension, and right and left rotation. The 25 degrees corresponds to the mid-range of the cervical spine, and all of the participants have unrestricted access to the middle of the range [28]. During cervical reposition sense assessments in therapeutic practice, we observed patients experienced a satisfactory level of comfort within this middle range. The subjects were instructed to sit on a chair and close their eyes for the JPS examination. The subject’s shoulder was fixed with the strap to avoid alternate movements during the JPS assessment. The CROM gadget is attached to the head of the individual using Velcro. The magnetic yoke is positioned around the subject’s shoulder such that its arrow points north. The target head position test is initiated by the examiner passively moving the subject’s head to 25 degrees of flexion, maintaining that position for five seconds, and asking the subject to remember it. The head is then returned to its neutral position. The patient was then instructed to actively move his neck and reposition himself in the target position. Once the subject is repositioned to the target position, he or she signaled by saying “yes”. The repositioning sense was assessed as JPE in degrees. Similar tests were conducted in the directions of cervical extension and right and left rotation. Six JPE test trials were conducted this way, and the mean of these trials was computed in the results.

### 2.4. Limits of Stability

**Figure 2:**
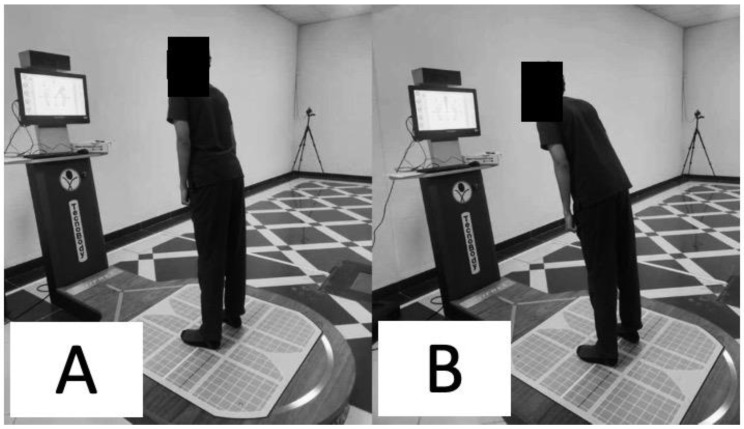
Limits of stability assessment (**A**) starting and (**B**) moving to a target.

The limits of stability were assessed using computerized dynamic posturography (Iso-free—Techno body, Bergamo, Italy). The limits of stability are the area over which an individual can move safely without altering the base of support. It consists of a circular platform and the following primary elements: a stabilometric posture platform, a touch screen, a 3D camera, and specific software. The stabilometric force platform analyses the center of pressure when the individual is in the standing posture sensing the pressure from the platform [29]. The individuals were asked to stand on the posturography force platform with both feet together in a standardized manner [29]. The subject was asked to look into the screen in front of them and follow the targets provided on the screen by the posturography device intended to assess the limits of the stability [29]. Only once did the target randomly appear in all eight directions, as shown on the screen in a blink. Without moving their feet, the individuals are told to shift their center of mass toward the objective (Figure 2). 

The instrument records and provides a score for the amount of sway needed to reach the target from the center along the exact shortest vertical or horizontal path. The limits of stability were assessed in eight directions: A1—forward, A2—forward right, A3—right, A4—right-backward, A5—backward, A6—left-backward, A7—left, A8—left-forward. A score of 100 is the maximum score that can be obtained in a direction. The computer calculates the limits of stability score for a particular test as a percentage of the ideal score, which is 100. A lower score indicated a more significant sway. The limits of stability parameters included are reaction time in seconds, maximum excursion in percentage, and directional control in percentage [30]. The reaction time is calculated as the ability of the individual reacting to reach the target position initiating the voluntary movement in response to a stimulus provided on the computer screen [30]. To determine the respondents’ maximal excursion, they were asked to lean as far as they could toward one of the eight randomly assigned spatial target places while maintaining 100% of their stability [30]. For the purpose of establishing directional control, the amount of movement of normalized COP in the target direction (in the direction that leads toward the target) is contrasted with the amount of movement in the off-target direction [30]. The overall on-target movement is shown in the formula: [(the percentage of on-the-target movement—the percentage of off-the-target movement)/(percentage of on-the-target movement)] × 100 [30]. 

The TSK sores, cervical JPE, and the limits of stability variables were recorded by the third-party examiner who was blinded to the groups.

### 2.5. Sample Size Determination

The key findings of the research served as the basis for determining the ideal number of participants in the sample, and the mean and standard deviation of the cervical JPE measurements served in this capacity [7]. To have 80% power to detect an effect size and a significance level of 0.05, we require a minimum of 45 participants in each group. To account for the possibility of missing data or dropouts, we enrolled 50 people in each group.

### 2.6. Statistical Analysis

**Figure 3:**
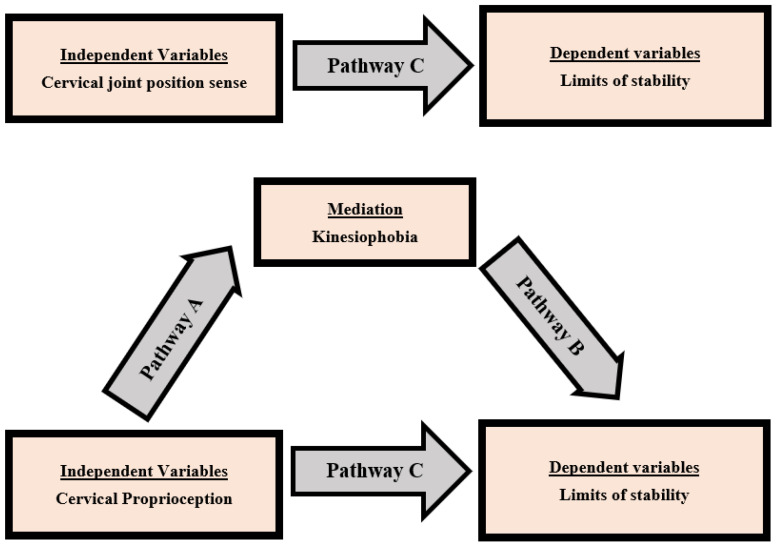
Model of the potential mediating effect of kinesiophobia on the relationship between cervical joint position sense and limits of stability.

The Shapiro–Wilk tests were used to assess the data distribution’s normality. The study variables are expressed in mean and standard deviation. Differences in cervical JPS and limits of stability characteristics between FMS and asymptomatic groups were analyzed using ANOVA. The cervical JPE and limits of stability were correlated using Pearson’s correlation coefficient (r). The correlation was estimated as low (r = 0.20 to 0.30), moderate (r = 0.31 to 0.69), and high (r = 0.70 to 1) [31]. A mediation analysis using multiple regression was computed with kinesiophobia as a mediator (Figure 3). 

We utilized bivariate regression to calculate the direct effect (a) between cervical JPS and kinesiophobia. Using multiple regression with kinesiophobia as a predictor and limits of stability as a dependent variable, the direct effect (b) between kinesiophobia and stability limits was estimated. Using multiple regression with kinesiophobia as the predictor and limits of stability as the dependent variable, one goal was to estimate the direct effect (c) between cervical JPS. The statistical analyses were carried out with version 22.0 of Statistical Package for the Social Sciences (SPSS) (IBM SPSS Statistics), and the level of statistical significance was established at *p* less than 0.005.

## 3. Results

**Figure 4:**
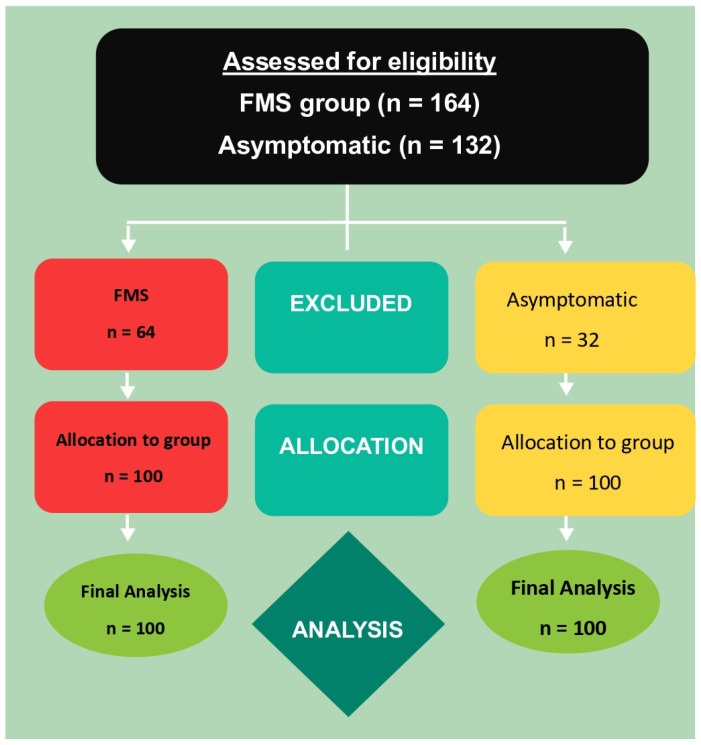
Consort flow of the cross-sectional study.

The assessment of eligibility of FMS and asymptomatic and consort flow of this cross-sectional study is summarized in Figure 4. 

The demographic characteristics of the study population are summarized in Table 1. On a VAS scale, the mean score for pain was 4.34, and the score for kinesiophobia was 42.44 (Table 1).

The FMS group individuals had impaired cervical JPS compared to the asymptomatic group (Table 2).

The magnitude of the mean cervical JPE in the FMS group was larger (flexion: F = 277.07, *p* < 0.001; extension: F = 265.57, *p* < 0.001; rotation right: F = 239.28, *p* < 0.001; rotation left: F = 233.43, *p* < 0.001) compared to the asymptomatic group. The mean cervical JPE in the FMS group ranged between 5.13° (rotation left) to 6.30° (extension) and 2.39° (flexion) to 3.05° (extension). The mean JPE in the cervical extension direction (FMS group: 6.30° ± 1.50°, asymptomatic group: 3.05° ± 1.31°) was larger in comparison with the other directions tested. 

**Figure 5:**
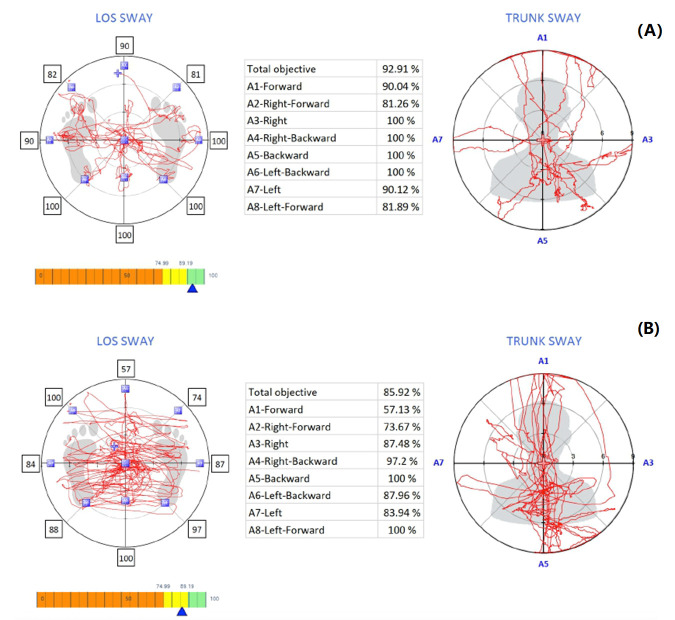
Limits of stability and trunk sway in (**A**) asymptomatic and (**B**) fibromyalgia syndrome individuals.

**Figure 6:**
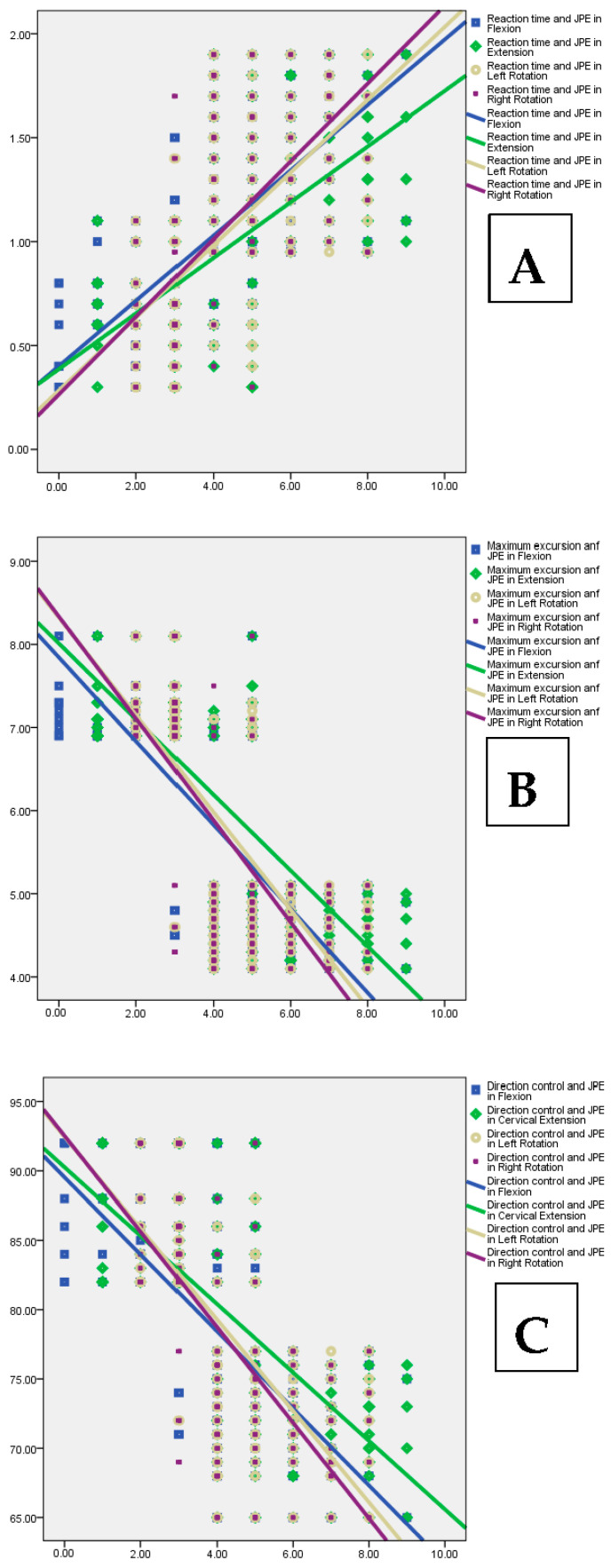
Correlation between cervical joint position errors and limits of stability tests: (**A**) reaction time (s); (**B**) maximum excursion (%); (**C**) direction control (%).

The limits of stability tests showed that FMS group individuals had a longer reaction time (1.40 ± 0.31 s) and reduced maximum excursion (4.59 ± 0.38) and direction control (71.66 ± 3.53) percentages. The LOS was significantly impaired in the FMS group (*p* < 0.001) with increased sway compared to the asymptomatic group (Table 2, Figure 5). Cervical JPS showed significant relationships with LOS tests (Figure 6, Table 3). 

Cervical JPE showed significant moderate-to-strong correlations with a reaction time (r = 0.58 ** to 0.64 **) and maximum excursion (r = −0.71 ** to −0.74 **) and direction control (r = −0.66 ** to −0.68 **) parameters of the LOS test. The results of the mediation analysis are summarized in Table 4 and Table 5. 

As shown in Figure 3, in this mediation model, the total effect was the observed effect of cervical JPS on limits of stability variables (reaction time, maximum excursion, and directional control) (pathway C). The total effect also decomposed into the direct effect of cervical JPS on limits of stability (pathway C′) and the indirect effects of JPS on limits of stability through the kinesiophobia (mediated: pathway A + B). The indirect effect was statistically significant (Sobel test) in that kinesiophobia explained the association between cervical JPS and limits of stability (*p* > 0.05).

## 4. Discussion

The results of this study showed that the magnitude of cervical JPS errors was larger and limits of stability was impaired in FMS individuals compared to asymptomatic. The cervical JPS showed a significant moderate-to-strong relationship with limits of stability variables. Moreover, kinesiophobia mediated the relationship between JPS and limits of stability in FMS individuals.

The results of this study showed that FMS individuals had an increased magnitude of cervical JPE compared to asymptomatic individuals. The reasons for altered cervical joint position sense can be attributed to the following reasons. The cervical spine is rich in mechanoreceptors and contributes to afferent proprioceptive input to the higher centers [14]. Patients with FMS may exhibit abnormal motor behavior in terms of timing and activation [32]; a decrease in the area of the muscles’ cross-section; and functional deficiencies in the muscles’ strength, endurance, precision, and accuracy [33]. The presence of neck pain can lead to maladaptive strategies, which can change the coordination of the muscles, as well as a decreased specificity of the activation of the muscles in the neck. This is demonstrated, for instance, by the decreased activation of deep segmental muscles and the increased activation of superficial muscles [34,35]. As was previously determined, the muscle spindles that are closely grouped in the deep neck muscles are the principal source of JPS afferents in the region of the neck [36]. Because of these morphological and functional changes in the cervical deep and superficial muscles, there is a possibility that the discharge of muscle spindles would shift. This will cause a change in the afferent input and will have an effect on the JPS [37]. The findings show that people who experience persistent neck pain also experience the intricacy and varied characteristics of weak neck muscles. Moreover, the effects of pain at different levels of the nervous system can cause differences in the sensitivity of muscle spindles and in the way the cortex reflects and regulates the information received from cervical afferents [38,39].

Cervical JPEs varied in magnitude from 5.13° to 6.30° in FMS participants in this study, and similar JPE magnitudes were found in CNP participants in earlier published investigations [19,28,37,40,41,42]. Studies that evaluated cervical JPS in different age groups showed that the magnitude of cervical JPE was larger in the elderly subjects compared to younger individuals [41,42,43]. Reddy et al. [41] conducted a study to assess the impact of CNP on cervical 337 JPS in elderly individuals aged 65 years or over and concluded pain significantly impacted the cervical proprioceptive sensibility [41]. Alahmari et al. [43] also stated that elderly individuals with CNP over 50 years have larger cervical JPE (6.57° to 7.80°) compared to age-matched asymptomatic individuals (1.95° to 2.95°). Our results potentially propose that CNP individuals are associated with decreased proprioceptive acuity.

Regarding the limits of stability, it quantifies movement traits related to participants’ volitional ability to modify their spatial position and their ability to stay stable in that posture [30]. The results of the current investigation on limits of stability in double stance support earlier findings that patients with FMS perform worse than healthy people in trials with both open and closed eyes when posturography is used to measure postural stability [44]. This finding substantiated the findings of prior research that showed a relationship between these neuromuscular variables and postural control in FMS patients [7,45,46]. Postural control may be impacted by the interplay of muscular weakening and proprioceptive inaccuracy [7]. Patients with muscle weakness have decreased muscle mass, incomplete muscle activation, decreased muscle spindle sensitivity, fewer sensory units, and fewer mechanoreceptors, all of which can have an effect on the precision of the JPS and limits of stability [33].

This study showed a strong relationship between cervical JPE and limits of stability variables. Larger proprioceptive errors are correlated with increased reaction time, slower maximum excursion, and direction control parameters. Gucmen et al. [17]. showed a significant correlation between sway velocities and cervical active repositioning tests (r = 0.51, *p* < 0.001). Moreover, Reddy et al. [7] showed a higher correlation (r = 0.75, *p* < 0.001) between the increased magnitude of cervical JPE and increased postural sway values in FMS individuals. These results and those of the current investigation show that cervical JPS has a more significant role in the regulation of dynamic standing balance than static, which is necessary for the prevention of falls. The cervical JPE and reaction time had a positive association (r = 0.56 to 0.64, *p* < 0.001), which suggests that FMS participants responded to a visual stimulus more slowly as the cervical JPS was significantly impaired. The reaction times of older participants were measured in a study that was carried out by Lord et al. [47]. The participants were asked to choose one of four targets and walk on it as quickly as they could when it was lighted. Further analysis of our findings revealed a negative relationship between the cervical JPE and the maximum excursion (−0.71 to −0.74, *p* < 0.001) and directional control (−0.66 to −0.68, *p* < 0.001) parameters. In other words, participants with better cervical position sensing acuity were able to increase their stability thresholds. Prior research has demonstrated that the limits of stability decreased with the degeneration process and was a key predictor of multiple falls [7,48]. When compared to those who had never fallen before, they discovered that fallers had significantly longer reaction times and lesser maximum excursion and direction control parameters. The current study generates a notion that increasing cervical JPS may improve reaction time and lessen the occurrence of falls among the elderly; this aspect must be examined in future studies.

The relationship between cervical JPS and limits of stability was fully mediated by kinesiophobia. Consequently, greater pain severity has a deleterious effect on motor function via increased fear of movement. These findings lend credence to the hypothesis that, within the parameters of the stability test, the psychological evaluation and response to potential injury and pain escalation because of movement may play an important role. This notion is supported by the fact that pain is a potent signal that demands one’s full attention and prompts one to take preventative measures in order to avoid or lessen its effects [49]. As a result, kinesiophobia may serve as a protective mechanism against discomfort and the worsening of pain by encouraging the avoidance of movement and the restriction of movement. It is probable, according to the Fear Avoidance Model, that a repertoire of protective cognitive and affective reactions, such as a dread of movement, leads to safety-seeking behavior such as activity avoidance. This is because the fear of movement is one of these reactions [50]. These protective behaviors are helpful in the case of acute pain because they avoid engagement with the pain. However, in the situation of chronic pain, this avoidance of interaction increases impairment.

However, tackling kinesiophobia in this demographic might have a health advantage. Lowering kinesiophobia may increase motor performance, improve proprioceptive acuity, and improve balance and stability limits. Overall, it indicates that fear of movement, chronic pain, proprioceptive, and balance should be primary priorities in the multidisciplinary treatment of persons with FMS. By decreasing kinesiophobia, the effect of JPS on limitations of stability can be mitigated.

It is essential to note that our findings provided support for the mediation of kinesiophobia, showing that the selected mediator was appropriate. Notwithstanding this, it is of the utmost importance to stress that the list of putative mediators is by no means complete. We did not consider evaluating catastrophic behavior, pain tolerance, or the severity of depressive symptoms [51]. In fact, pain tolerance and catastrophic behavior have a substantial effect on JPS and limits of the stability [52,53]. Moreover, depression and pain catastrophizing are two overlapping and interrelated phenomena [54,55]. Moreover, kinesiophobia and pain tolerance appear to be related to depression [56]. The incorporation of these variables into future models of mediation could result in unique and intriguing conclusions.

### Limitations

The study’s cross-sectional nature precludes us from drawing causal conclusions. In addition, potential confounding variables such as pain catastrophizing, anxiety, pain acceptance, and depression were not evaluated. With chronic pain syndromes such as fibromyalgia, where pain varies significantly between and within days, it may not be optimal to use a single assessment. An ecological momentary assessment design could provide valid data.

## 5. Conclusions

The cervical JPS and limits of stability are significantly impaired in FMS individuals compared to asymptomatic. The JPS showed a significant relationship with limits of stability. Kinesiophobia significantly mediated the relationship between JPS and limits of stability in FMS individuals. Psychological therapies that target these characteristics should be incorporated into interdisciplinary rehabilitation programs for FMS patients and proprioceptive acuity and limits of stability can be considerably enhanced by lowering kinesiophobia.

## Figures and Tables

**Table 1:** Demographic characteristics and physical parameters of the FMS and asymptomatic individuals.

Variables	FMS Individuals (*n* = 100)	Asymptomatic (*n* = 100)	*p*-Value
Age (years)	59.53 ± 7.72	58.30 ± 4.23	<0.001
Gender (M:F)	59:41	52:48	
Height (meters)	1.68 ± 0.09	1.73 ± 0.05	0.002
Weight (kg)	71.24 ± 5.96	69.58 ± 5.24	0.030
BMI (kg/m^2^)	23.50 ± 3.80	23.38 ± 2.74	<0.001
VAS score	4.84 ± 1.73	-	-
TSK score	41.44 ± 3.17	-	-

BMI = body mass index, VAS = visual analogue scale, TSK = Tampa scale of kinesiophobia.

**Table 2:** Descriptive characteristics of cervical joint position sense and limits of stability tests.

Variables	Above 50 Years (*n* = 100)	Below 50 Years (*n* = 100)	F	*p*-Value
JPE in flexion (°)	5.39 ± 1.29	2.39 ± 1.26	277.07	<0.001
JPE in extension (°)	6.30 ± 1.50	3.05 ± 1.31	265.57	<0.001
JPE in right rotation (°)	5.38 ± 1.38	2.95 ± 0.76	239.28	<0.001
JPE in left rotation (°)	5.13 ± 1.35	2.87 ± 0.61	233.43	<0.001
Reaction time (s)	1.40 ± 0.31	0.71 ± 0.29	128.74	<0.001
Maximum excursion (%)	4.59 ± 0.38	7.16 ± 0.32	976.75	<0.001
Direction control (%)	71.66 ± 3.53	85.80 ± 3.85	396.49	<0.001

JPE = joint position error; *p*-values are based on post hoc Bonferroni correction.

**Table 3:** Relationship between cervical joint position errors and balance and limits of stability tests (𝑛 = 100).

Variables	Reaction Time (s)	Maximum Excursion (%)	Direction Control (%)
JPE in flexion (°)	0.64 **	−0.74 **	−0.68 **
JPE in extension (°)	0.59 **	−0.73 **	−0.66 **
JPE in right rotation (°)	0.58 **	−0.72 **	−0.67 **
JPE in left rotation (°)	0.56 **	−0.71 **	−0.66 **

JPE = joint position error, ** = correlation is significant at the 0.01 level (two-tailed).

**Table 4:** Kinesiophobia as a mediation factor on the relationship between cervical JPS and limits of stability.

Explanatory Variables	Total Effect: Direct and Indirect			Direct Effect			Indirect Effect		
	B	SE	*p*-Value	B	SE	*p*-Value	B	SE	*p*-Value
Kinesiophobia × JPE in flexion (°) × reaction time	0.43	0.03	<0.001	0.42	0.04	<0.001	0.07	0.02	0.003
Kinesiophobia × JPE in extension (°) × reaction time	0.53	0.02	<0.001	0.51	0.03	<0.001	0.06	0.02	0.004
Kinesiophobia × JPE in left rotation (°) × reaction time	0.47	0.05	<0.001	0.38	0.05	<0.001	0.09	0.03	0.003
Kinesiophobia × JPE in right rotation (°) × reaction time	0.54	0.05	<0.001	0.37	0.05	<0.001	0.08	0.03	0.004
Kinesiophobia × JPE in flexion (°) × maximum excursion	0.49	0.05	<0.001	0.41	0.04	<0.001	0.07	0.02	0.002
Kinesiophobia × JPE in extension (°) × maximum excursion	0.58	0.03	<0.001	0.53	0.03	<0.001	0.06	0.02	0.003
Kinesiophobia × JPE in left rotation (°) × maximum excursion	0.47	0.05	<0.001	0.41	0.05	<0.001	0.09	0.03	0.004
Kinesiophobia × JPE in right rotation (°) × maximum excursion	0.53	0.03	<0.001	0.47	0.05	<0.001	0.08	0.03	0.003
Kinesiophobia × JPE in flexion (°) × direction control	0.44	0.05	<0.001	0.43	0.04	<0.001	0.07	0.02	0.001
Kinesiophobia × JPE in extension (°) × direction control	0.56	0.03	<0.001	0.53	0.03	<0.001	0.06	0.02	0.001
Kinesiophobia × JPE in left rotation (°) × direction control	0.49	0.05	<0.001	0.38	0.05	<0.001	0.09	0.03	0.003
Kinesiophobia × JPE in right rotation (°) × direction control	0.56	0.03	<0.001	0.57	0.05	<0.001	0.08	0.03	0.002

TSK = Tampa scale of kinesiophobia, B = unstandardized coefficients, SE = standard error.

**Table 5:** Sobel test for indirect effect for statistical significance.

Explanatory Variables	Sobel Test	SE	*p*-Value
Kinesiophobia × JPE in flexion (°) × reaction time	0.49	0.12	0.032
Kinesiophobia × JPE in extension (°) × reaction time	0.46	0.23	0.030
Kinesiophobia × JPE in left rotation (°) × reaction time	0.54	0.32	0.038
Kinesiophobia × JPE in right rotation (°) × reaction time	0.67	0.19	0.024
Kinesiophobia × JPE in flexion (°) × maximum excursion	0.42	0.26	0.053
Kinesiophobia × JPE in extension (°) × maximum excursion	0.69	0.36	0.026
Kinesiophobia × JPE in left rotation (°) × maximum excursion	0.52	0.38	0.038
Kinesiophobia × JPE in right rotation (°) × maximum excursion	0.68	0.18	0.024
Kinesiophobia × JPE in flexion (°) × direction control	0.51	0.19	0.030
Kinesiophobia × JPE in extension (°) × direction control	0.62	0.31	0.020
Kinesiophobia × JPE in left rotation (°) × direction control	0.74	0.16	0.018
Kinesiophobia × JPE in right rotation (°) × direction control	0.66	0.91	0.024

SE = standard error.

## Data Availability

All data generated or analyzed during this study are included in the Supplementary Information Files.

## Supplementary Materials

The following supporting information can be downloaded at: https://www.mdpi.com/article/10.3390/jcm12082791/s1, Table S1: Study Data.
